# Research Progress on Anti-Hyperlipidemia Peptides Derived from Foods

**DOI:** 10.3390/nu17071181

**Published:** 2025-03-28

**Authors:** Mingxia Zhao, Kaina Qiao, Lili Zhang, Li Liang, Shuxing Chen, Lishui Chen, Yuyu Zhang

**Affiliations:** 1 Food Laboratory of Zhongyuan·Beijing Technology and Business University, Luohe 462300, China; 2 Food Laboratory of Zhongyuan, Luohe Food Engineering Vocational University, Luohe 462000, China; 3 Key Laboratory of Geriatric Nutrition and Health, Ministry of Education, Beijing Technology and Business University, Beijing 100048, China; 4 Key Laboratory of Flavor Science of China General Chamber of Commerce, Beijing Technology and Business University, Beijing 100048, China; 5 Food Laboratory of Zhongyuan, Luohe 462300, China

**Keywords:** hyperlipidemia, peptides, mechanism of action, foods

## Abstract

Hyperlipidemia is a metabolic disorder in which cholesterol (TC) and triglycerides (TGs) in the blood exceed the normal physiological levels. The incidence of the condition has continued to rise in recent years, posing a serious threat to public health. Its clinical treatment mainly relies on drug interventions, such as statins, fibrate, and niacin. Although these drugs have shown some efficacy in the treatment of hyperlipidemia, their adverse effects cannot be ignored. In contrast, naturally derived peptides have gradually become potential candidates for the prevention and treatment of hyperlipidemia due to their strong anti-hyperlipidemic activity and safety; examples of such peptides include those from dairy products, grains, legumes, and seafood. This review systematically summarizes peptides with anti-hyperlipidemic activity and analyzes their mechanisms of action, providing a theoretical basis for further research. In addition, we also outline some challenges facing the application of peptides, hoping to prevent hyperlipidemia and reduce its incidence by encouraging the consumption of foods rich in anti-hyperlipidemia peptides.

## 1. Introduction

Hyperlipidemia, also referred to as dyslipidemia, is a metabolic disorder characterized by abnormal levels of cholesterol (TC) and triglycerides (TGs) in the blood. Hyperlipidemia is closely related to the occurrence and development of cardiovascular and cerebrovascular diseases such as atherosclerosis, coronary heart disease, and cerebral stroke. The global incidence of hyperlipidemia is steadily increasing, posing a significant public health concern [1]. Epidemiological studies of hyperlipidemia have shown that its incidence is closely related to factors such as age, sex, genetics, dietary habits, lifestyle, and socioeconomic status [2]. A decline in organ function, particularly in the liver and pancreas, can impair lipid metabolism, leading to abnormal blood lipid levels. The pathophysiological basis of hyperlipidemia is the disorder of lipid metabolism. Lipid metabolism in the human body involves the synthesis, uptake, transport, and excretion of lipids. When one or more of the above processes are abnormal, the abnormal accumulation of lipid components in the blood occurs [3], resulting in hyperlipidemia. In addition, the disorder of lipid metabolism also interacts with pathological processes such as inflammatory response, oxidative stress, and abnormal platelet function [4]. Genetic factors also play an important role in the development of hyperlipidemia. Familial hypercholesterolemia (FH) is a common genetic disorder of abnormal lipid metabolism caused by mutations in the low-density lipoprotein receptor (LDLR) gene, resulting in significantly elevated levels of low-density lipoprotein cholesterol (LDL-C) [5]. Dietary habits, especially diets that are high in fat, cholesterol, sugar, and salt, as well as poor lifestyles such as lack of exercise, obesity, smoking, and excessive alcohol consumption, are important factors leading to dyslipidemia [6]. Currently, the diagnosis of hyperlipidemia mainly relies on blood biochemical tests, including the determination of indicators such as TC, LDL-C, HDL-C, and TGs [7].

The therapeutic goal of hyperlipidemia is to reduce the level of lipids in the blood and to reduce the risk of cardiovascular and cerebrovascular events. Treatment strategies include the modification of lifestyle and pharmacotherapy. Specifically, lifestyle adjustments are fundamental to the treatment of hyperlipidemia, including reasonable diet, moderate exercise, weight control, and smoking cessation [8]. Drug therapy includes the use of statins, fibrates, niacin, and TC absorption inhibitors. Although pharmacological interventions are effective, most of them have adverse side effects [9]. Naturally derived peptides have high anti-hyperlipidemic activity and strong safety profiles; these have become candidates for preventing and ameliorating hyperlipidemia. Polypeptides are compounds made of two or more amino acids linked by peptide bonds, resulting from the breakdown of proteins. Recently, peptides have shown great potential in the fight against hyperlipidemia [10,11].

This review explores the inducing factors of hyperlipidemia, the mechanism of action, and the source of anti-hyperlipidemia peptides to provide a theoretical basis for the study of polypeptides in the field of anti-hyperlipidemia. Future research should further explore foods that are rich in anti-hyperlipidemic peptides, as well as their mechanisms of action, providing new strategies and methods for the treatment of hyperlipidemia. Moreover, we also discuss the bioavailability, stability, clinical application, and future research directions of peptides.

## 2. Predisposing Factors of Hyperlipidemia

### 2.1. Gene Expression

FH is mainly characterized by a significant increase in the concentration of LDL-C in the blood. This abnormally elevated LDL-C level can cause extravascular cholesterol deposition, triggering a range of clinical manifestations such as tendon xanthoma, cutaneous xanthoma, corneal arch, and intravascular cholesterol deposition. These deposits are the main pathological basis of atherosclerosis. Due to the presence of FH, patients often show the progression of atherosclerosis, as well as early-onset coronary artery disease (CHD), which significantly increases the incidence and mortality of cardiovascular disease. Initially, FH was considered to be an “essential” form of type IIa hyperlipoproteinemia, which is associated with pathogenic variations in the LDLR gene (often referred to as “mutations”). However, it has been found that defects in other genes can also lead to elevated LDL-C levels and the emergence of the FH phenotype. Therefore, the term “FH” is currently commonly used to refer to all forms of type IIa hyperlipoproteinemia, including autosomal dominant hypercholesterolemia (ADH) and autosomal recessive hypercholesterolemia (ARH). Together, these different genetic forms constitute the complex spectrum of the inherited FH disease [12].

As one of the ligands of LDLR, apolipoprotein B (APOB) plays a key role in cardiovascular diseases such as lipid metabolism and atherosclerosis. Although the existence of APOB in very-low-density lipoprotein (VLDL) and intermediate-density lipoprotein (IDL) has been confirmed, its mechanism of action in LDL still needs further investigation. APOB in plasma exists in two forms—APOB48 and APOB100. APOB48 is mainly synthesized in the small intestine [13], while APOB100 is mainly synthesized in the liver [14]. The major difference between APOB100 and APOB48 lies in the C-terminal sequence [15], which is the key region for LDLR binding. Most of the reported APOB gene variants cause inactivating variants with hypolipoproteinemia. However, incorrect variants can lead to ADH [16]. Studies have indicated that the autosomal dominant disorder FH is characterized by elevated LDL-C levels due to the reduced clearance of APOB by hepatocytes; further studies have stated that the defect is not related to the LDLR, but rather to the LDL itself [17,18].

In 2009, Abifadel et al. identified a mutation in the proprotein-converting enzyme subtilisin 9 (PCSK 9), which is located on human chromosome 1 p32.3, in FH patients. Loss-of-function mutations in PCSK 9 are associated with lower LDL-C levels and the incidence of coronary heart disease, whereas gain-of-function mutations cause the overexpression of protein function and are associated with an increased risk of FH [19]. PCSK 9, also known as neural cell apoptosis regulator convertase-1, is a soluble secreted serine protease composed of 692 amino acids with a molecular weight of 72 kD [20]. The structure of mature PCSK 9 includes the signal sequence, the prodomain, the catalytic domain, and the C-terminal domain, which contains cysteine and histidine [21]. PCSK 9 is secreted by the liver, and its concentration depends on its own synthesis, processing, and clearance. The main function of secreted PCSK 9 is to regulate the amount of LDLR on the cell surface [22]. Before explaining the physiological role of PCSK 9, the clearance and catabolic process of LDL-C should be clarified. In plasma, LDL-C binds to the LDLR and forms a complex, which enters cells through grid-mediated endocytosis; this is then degraded and the LDLR returns to the membrane surface of the hepatocytes and enters the next round of circulation [22]. The catalytic domain of PCSK 9 is able to bind at the cell membrane to the *N* terminus of the first epidermal growth factor-like repeat domain A (EGF-A) of the LDLR; this is a step that prevents LDLR from recycling to the cell surface, thereby enhancing lysosomal degradation. Moreover, PCSK 9 also enhances LDLR degradation through a relatively fast intracellular pathway [23]. Through the above effects of PCSK 9, LDL-C levels in plasma increased. In 2018, it was shown that LP(a) levels decrease after the inhibition of PCSK 9, possibly due to the decreased secretion of LP(a)-apo(a), thus decreasing the synthesis of LP(a), while also increasing the uptake and clearance of LP(a) by LDLR [24].

### 2.2. Eating Habits

Predisposing factors that can lead to hyperlipidemia also include unhealthy eating habits. Specifically, the long-term intake of high fat, high-cholesterol foods, such as fried food and animal viscera, could cause obesity, leading to an increase in the lipid content in the blood, eventually causing hyperlipidemia [25]. In 2022, studies showed that elevated levels of TGs and TC are proportional to the degree of obesity. In addition, the intake of foods that are rich in sugar and refined carbohydrates, such as sweet drinks, pastries, and white bread, significantly promotes the synthesis of TGs in the human body [26]. These high-sugar foods are quickly converted into glucose in the body, leading to a sharp rise in blood sugar levels. In response to elevated blood glucose, insulin is secreted to promote the conversion of glucose into energy or storage [27]. However, when sugar and refined carbohydrates are overconsumed, the secretion of insulin increases accordingly, which promotes the liver to convert excess glucose into TGs. Moreover, the accumulation of TGs in the body can lead to elevated lipid levels and can increase the risk of cardiovascular disease [28]. In 2022, high-sodium diets were shown to cause elevated blood pressure, which increases the risk of cardiovascular and cerebrovascular diseases, such as hyperlipidemia. As the main component of salt, an increase in sodium intake will lead to an increase in sodium concentration in the blood, which will increase the retention of water in the body, leading to the expansion of blood volume, and eventually causing an increase in blood pressure. Long-term hypertension will cause persistent damage to the vascular wall, resulting in a reduction in vascular elasticity and facilitating the formation of arteriosclerosis. Arterial stiffness, as one of the important risk factors for cardiovascular disease, can significantly increase the risk of heart disease and stroke [29].

### 2.3. Lifestyle Habits

Poor lifestyle, such as lack of exercise, insufficient sleep, smoking, and excessive drinking, is an important factor leading to dyslipidemia. Insufficient exercise can cause fat metabolism disorders in the body, thus increasing the risk of hyperlipidemia [30]. Several studies have revealed that regular aerobic exercise can effectively reduce the level of LDL-C and increase the level of HDL-C, thereby optimizing the blood lipids. In addition, insufficient exercise can also lead to the accumulation of TGs, which are one of the lipid indicators associated with an increased risk of cardiovascular disease [31,32]. Insufficient physical activity can also negatively affect insulin sensitivity and may contribute to the emergence of insulin resistance [33], which is a key risk factor for type 2 diabetes. It should be noted that diabetic patients are usually also diagnosed with dyslipidemia. In addition, lack of exercise can slow down the speed of metabolism and reduce the efficiency of fat decomposition and energy consumption in the body, thus further exacerbating hyperlipidemia [34].

Sleep deprivation could lead to an imbalance in hormone levels, especially hormones that control hunger, such as insulin, leptin, and ghrelin [35]. When sleep is scarce, insulin sensitivity decreases, leptin levels fall, and ghrelin levels rise, which increases appetite and prompts the body to consume more high-calorie foods [36]. In addition, sleep deprivation also affects the body’s stress response, leading to increased cortisol levels that promotes the accumulation of fat, especially abdominal fat, thus increasing the risk of hyperlipidemia [37].

Smoking can cause an increase in LDL-C levels in serum, while decreasing HDL-C levels [38]. LDL-C tends to deposit on the blood vessel walls to form plaques, while HDL-C helps to clear cholesterol from the blood vessels [39]. Smoking can break this balance by affecting lipoprotein metabolism [40]. Smoking is also associated with increased serum TG levels, which increase TG levels in the blood by affecting the breakdown of adipose tissue and the synthesis of TGs in the liver; high TG levels are also associated with atherosclerosis and insulin resistance. Tobacco smoke contains large amounts of free radicals, which attack lipid molecules, resulting in lipid peroxidation; these then damage the integrity of cell membranes, affecting the normal metabolism of lipids. Smoking also weakens the body’s antioxidant defense system and reduces the activity of antioxidant enzymes such as superoxide dismutase (SOD) and glutathione peroxidase (GPx), which can lead to the intensification of oxidative stress, further promoting lipid peroxidation and increasing the risk of atherosclerosis. In 2020, a study showed that smoking induces the release of inflammatory cytokines in the body, such as tumor necrosis factor α (TNF-α) and interleukin 6 (IL-6). These inflammatory factors can encourage the liver to synthesize more VLDL, which is converted to LDL in the blood, thus increasing the levels of LDL-C; however, this also causes vascular endothelial dysfunction, affecting the normal function of vasodilation and vasoconstriction. Endothelial dysfunction is closely associated with the formation of atherosclerosis, further increasing the risk of cardiovascular disease. Smoking can interfere with insulin signaling, leading to insulin resistance, and has been linked to metabolic syndrome, which includes hyperlipidemia. In the state of insulin resistance, the amount of fatty acids in the liver decreases and the release of fatty acids increases, which subsequently promotes VLDL synthesis and secretion. VLDL is converted to LDL in the blood, increasing the levels of LDL-C while reducing the generation of HDL-C [41].

## 3. The Anti-Hyperlipidemia Mechanism of Peptides

### 3.1. Inhibition of the Synthesis of Cholesterol

There are two main sources of TC in the human body—ingestion through food and synthesis by the liver [42]. Acetoacetyl-coenzyme A (acetyl CoA) is a direct raw material for the synthesis of cholesterol in the liver, which comes from the metabolites of glucose, fatty acids, and certain amino acids. In addition, an adenine nucleoside triphosphate (ATP) energy supply and a reduced coenzyme (NADPH) hydrogen supply are required [43]. The synthesis of 1 TC requires 18 acetyl CoA, 36 ATP, and 16 NADPH molecules. The TC synthesis process is more complex, with nearly 30 reaction steps; the whole process is based on the following three stages: (1) the generation of 3-hydroxy-3-methyl glutarate monoacyl coenzyme A (HMGCoA) [44]; (2) the generation of mevalonic acid (MVA) [45]; and (3) the production of TC, whereby MVA first undergoes phosphorylation, decarboxylation, and dehydroxylation, before undergoing condensation to produce 30C. Squalene produces lanolin sterol via ER cyclase and oxygenase, and the latter loses three C and synthesized 27C TC [46].

**Figure 1:**
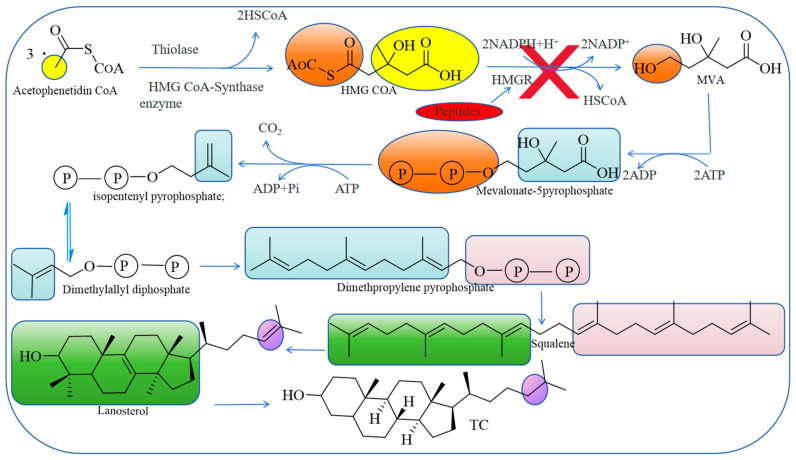
Peptides inhibit the synthesis of cholesterol.

In 2019 and 2007, researchers found that peptides competitively inhibited HMGR activity by mimicking the three-dimensional structure of HMG-CoA, allowing them to tightly bind to its active site [47,48]. This binding mode effectively hinders the HMGR-catalyzed reaction because the peptides occupy the active center of HMGR, making HMG-CoA unable to bind to HMGR, thus preventing cholesterol synthesis and reducing cholesterol levels in the blood (Figure 1). This inhibition is reversible, potentially reducing the risk of long-term side effects compared to traditional drugs [49,50]. Compared with traditional irreversible inhibitors, peptides can quickly restore the normal function of the enzyme after stopping its usage and reduce the adverse reactions that may be caused by long-term medication due to their reversibility, which has advantages for patients with hypercholesterolemia who need long-term management. This characteristic of peptides makes them a widely used clinical treatment and has provided new ideas and methods for the treatment of hypercholesterolemia.

### 3.2. Promotion of the Excretion of Cholesterol

**Figure 2:**
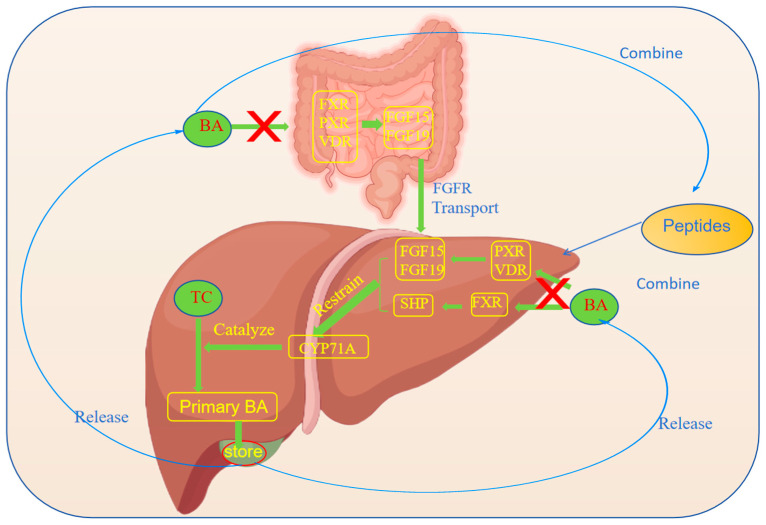
Peptides promote the excretion of cholesterol.

In recent years, several studies have revealed the significant role of peptides in promoting the excretion of TC (Figure 2). TC is mainly excreted through bile acid (BA) in the human body, a function that is mainly performed in the liver, where TC is converted into primary BA and is subsequently secreted into the gallbladder for storage [51]. When foods enter the small intestine, the gallbladder releases BA to facilitate fat digestion. In the lower segment of the small intestine, most of the BA is reabsorbed through the hepatic circulation of bile. A part of the BA is excreted with the bile, and a small part of the BA is excreted by intestinal bacteria [52,53]. The liver can also directly discharge TC into the intestine or through the intestinal mucosa [54], and TC can also be reduced to fecal sterol [55]. In 2019, Ticho et al. found that peptides, by mimicking the structure of BA, bind to receptors on liver cells to activate the signaling pathway. Studies have shown that BA directly binds to and activates four receptors—the farnesoid X receptor (FXR), the vitamin D receptor (VDR), the pregnane X receptor (PXR), and the constitutive androstane receptor (CAR) [56]. BA-activated receptors in the liver and/or intestine regulate the expression of proteins involved in the liver/or intestine that are normally exposed to relatively high concentrations of BA, where FXR activation induces the secretion of fibroblast growth factor 15 (FGF15) and fibroblast growth factor 19 (FGF19). FGF15 and FGF19 are transported to the liver via the FGF receptor (FGFR) to inhibit BA metabolism [57]. The activation of hepatic FXR also reduces BA synthesis by upregulating the expression of the transcriptional repressor small heterodimer ligand (SHP) [58]. In these tissues, these receptors act as sensors of BA levels in enterohepatic circulation and help to maintain BA homeostasis by regulating the genes involved in BA synthesis, transport, and detoxification [59]. The organism has two BA synthesis pathways, including the classical pathway (mainly producing 12α-hydroxyl BA and Chic acid) and the alternative pathway (mainly producing non-12α-hydroxyl BA and chenodeoxycholic acid). The process mainly involves the key enzymes for BA synthesis, such as cholesterol 7α-hydroxylase (CYP7A1), which is the rate-limiting enzyme in the BA synthesis pathway and is the first direct target of the liver cell receptor [60]. CYP7A1 and CYP 27 are the rate-limiting enzymes of the two pathways, and the synthesis of the former accounts for more than 70% of the total synthesis [61]. In addition, PXR and VDR inhibit CYP7a1 through the FGF15/FGF19 pathway [62], and peptides can indirectly promote the activity of CYP7A1 by affecting signaling molecules such as AMP-activated protein kinase (AMPK) or the insulin signaling pathway [63]. The activation of hepatic AMPK leads to the increased oxidation of fatty acids and the inhibition of hepatic TC synthesis. Thus, the expression of the CYP7A1 gene is important for maintaining the balance between TC and BA. In addition, peptides may also combine with BA [64], block the enterohepatic circulation of BA, increase the excretion of BA, and indirectly promote the transformation of TC into BA in the liver [65]. Studies have shown that peptides affect the enterohepatic circulation of BA by regulating the composition of intestinal microbiota, which then impacts the secretion of BA [66]. Specific peptide sequences and structural characteristics are important to stimulate the secretion of BA [67].

### 3.3. Regulation of the Lipoprotein Metabolism

**Figure 3:**
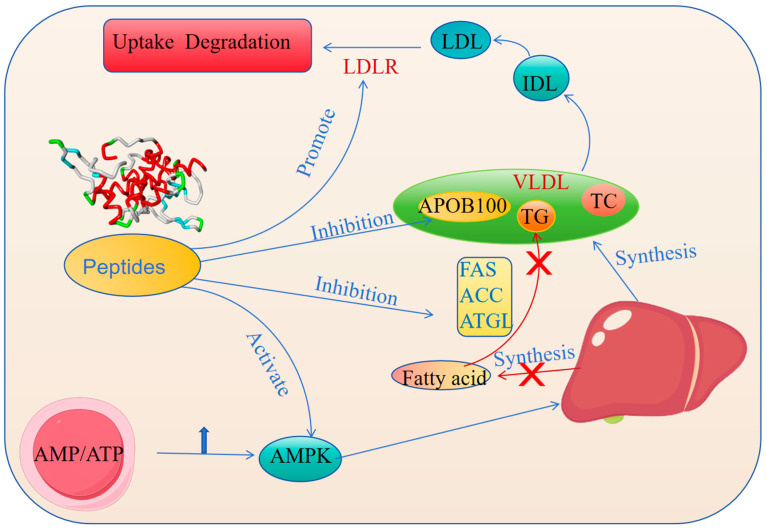
Peptides regulate lipoprotein metabolism.

Lipoproteins play a crucial role in the metabolism process of the human body, and they are responsible for transporting fat and TC throughout the body for cells to use or store. However, the dysregulation of lipoprotein metabolism may lead to multiple cardiovascular diseases, such as atherosclerosis and coronary heart disease [68]. Lipoproteins are the complexes of lipids and proteins; they are divided into four categories according to their density and size—HDL, LDL, VLDL, and IDL. HDL is often referred to as “good cholesterol” because of its ability to transport cholesterol from the blood vessel wall to the liver, thereby reducing the risk of atherosclerosis. In contrast, LDL is called “bad cholesterol” because high levels of LDL tend to deposit on the blood vessel walls, forming plaques and increasing the risk of cardiovascular disease [69]. In recent years, peptides have been found to play a unique role in regulating lipoprotein metabolism, providing new ideas for treating related diseases. Peptides can regulate the synthesis and secretion of lipoprotein by binding to receptors on the cell surface of the liver [70]. During the synthesis of VLDL, components such as TGs, TC, phospholipids, and ApoB100 are assembled into VLDL particles and secreted into the blood through the Golgi apparatus [71]. Peptides can inhibit the synthesis of fatty acids in the liver, thereby reducing TG production [72]. A reduction in fatty acid synthesis can directly affect VLDL synthesis because of the higher TG content in VLDL particles. On the one hand, peptides are able to regulate the expression of ApoB100 [73], which is the major structural protein of VLDL granules and its expression level directly affects the synthesis and secretion of VLDL [74]. Peptides subsequently inhibit the generation of VLDL by affecting the synthesis and stability of ApoB100 [75]. On the other hand, peptides can regulate the activity of enzymes related to lipid metabolism, such as fatty acid synthase (FAS), acetyl-CoA carboxylase (ACC), and triglyceride lipase (ATGL). By modulating the activity of these enzymes, peptides can reduce the synthesis of VLDL, thereby inhibiting the generation of VLDL [76]. AMPK is an energy sensor that senses the energy state of the cells. AMPK is activated when the intracellular ratio of AMP/ATP increases, which subsequently suppresses fatty acid synthesis and promotes fatty acid oxidation [77]. In 2021 and 2019, investigators found that peptides activate AMPK and reduce fatty acid synthesis, thereby inhibiting VLDL synthesis [78,79]. Since LDL is derived from the metabolic transformation of VLDL in the blood, the inhibition of VLDL synthesis directly reduces LDL generation. The LDL receptor is the protein on the cell surface that is responsible for recognizing and binding to LDL particles in the blood, thereby facilitating the uptake and degradation of LDL [80]. Peptides are able to promote the expression of the LDL receptor and increase LDL clearance, thereby reducing LDL levels (Figure 3) [81].

### 3.4. Anti-Inflammatory and Antioxidative Effects

Hyperlipidemia is closely related to vascular endothelial inflammation and oxidative stress [82]. The anti-inflammatory and antioxidative effects of the peptides help to reduce vascular inflammation and protect vascular endothelial function, thereby reducing the risk of cardiovascular disease. Nuclear factor kappa-B (NF-κB), a transcription factor located in the nucleus, is responsible for regulating gene expression and plays a crucial role in the physiological process of the inflammatory response [83]. The inhibitor of NF-κB (IκB), an inhibitory protein of NF-κB, is present in the cytoplasm under normal physiological conditions. However, when cells are stimulated by external stimuli such as cytokines, bacterial products, viruses, and ultraviolet light, the IκB kinase (IKK) complex is activated, leading to the phosphorylation and ubiquitination of the IκB protein, which then triggers its degradation [84]. This process releases NF-κB, allowing it to migrate into the nucleus and activate transcriptional processes in a range of target genes. In 2019, peptides were found to interact directly with the IKK complex to inhibit its kinase activity, thereby blocking the phosphorylation and degradation of IκB. Peptides competitively bind to the IKK complex to inhibit its activity. In addition, peptides can also exert their effects by inhibiting the upstream signaling pathways of NF-κB activation, e.g., TNF-α binding to its receptor or TNF-α receptor-associated signaling molecules, thus blocking the activation of NF-κB. In addition to inhibiting the release of NF-κB, peptides can also directly bind to NF-κB, preventing its binding to DNA, and subsequently inhibiting the transcription of target genes. These peptides typically mimic specific sequences on DNA and competitively bind to NF-κB, reducing its transcriptional activity [79]. Antioxidant enzymes are a class of enzymes that can remove free radicals in the body, which are considered to be one of the main factors leading to cell damage and aging. Superoxide dismutase (SOD) and glutathione peroxidase (GSH-Px) are two important antioxidant enzymes that play a key role in the maintenance of the intracellular redox balance. SOD is able to convert superoxide anion free radicals into oxygen and hydrogen peroxide, reducing damage to cells. GSH-Px further protects cells from oxidative stress by catalyzing the reaction of reduced glutathione (GSH) and peroxide, reducing the peroxide to harmless alcohols [85]. It has been shown that peptides have the potential to directly or indirectly activate antioxidant enzymes to enhance their catalytic efficiency by interacting with the active site. Moreover, the peptides may also promote the expression of antioxidant enzyme genes by modulating intracellular signaling pathways, thereby increasing their synthesis. Peptides enhance the antioxidant capacity of cells by increasing the levels of intracellular antioxidants, such as GSH [86].

## 4. Sources of Anti-Hyperlipidemic Peptides

### 4.1. Marine Anti-Hyperlipidemic Peptides

Marine sources of bioactive peptides include fish, shellfish, crustaceans, and seaweed (Figure 3). In recent years, studies have found that peptides in the ocean have significant potential to fight high levels of blood lipids; they regulate blood lipid levels through various mechanisms, providing new ideas and methods for the prevention and treatment of hyperlipidemia. Marine bioactive peptides are usually prepared via the enzymatic hydrolysis of a variety of marine resources in vitro, generally including the single-enzyme method and the method involving two or more protease complex enzymes. At present, the main extraction methods of marine peptides include ultrafiltration, gel filtration chromatography, ion exchange chromatography, and reverse-phase high-performance liquid chromatography [87].

Sea cucumber is a valuable marine organism that contains bioactive peptides with various biological functions. Studies have shown that sea cucumber contains more than 50% of the gonad protein. In view of the high protein content of sea cucumber gonads and sea cucumber gonad hydrolysate (SCGH), the data showed that after 8 weeks of SCGH administration (Table 1), dyslipidemia was improved in mice, and the levels of TGs, TC, and LDL-C were reduced, indicating that SCGH had a hypolipidemic effect, which may be related to the hypolipidemic peptides it contains. Further studies have revealed that aliphatic amino acids, such as Gly, Ala, Val, Ile, and Leu, can remove BA by forming stable complexes with hydrophobic bonds [88].

Certain algae have been found to produce peptides with lipid-lowering properties. For example, two bioactive peptides—P1 (LDAVNR; 686 Da) and P2 (MMLDF; 655 Da; from spirulina enzymes)—had a preventive effect against initial vascular disease [89]. Shih et al. demonstrated that the green algae 11-peptide is a biomolecule with the potential to prevent chronic-inflammation-associated vascular diseases [90]. The platelet-activated factor acetylhydrolase (PAF-AH) inhibitory peptide derived from red algal *Palmaria palmata* had a good effect against arteriosclerosis [91]. A new peptide, isolated from the microalgae Isochrysis zhanjiangensis, had anti-apoptotic and anti-inflammatory effects on oxidized low-density lipoprotein (ox-LDL) and induced the improvement of arteriosclerosis in human umbilical vein endothelial cells (HUVECs) [92]. C-phycocyanin isolated from spirulina reduced the levels of TC, HDL, and TGs in rats and rabbits [117].

Fish proteolytic products reduced plasma TC levels and increased the concentration of HDL-C, as well as reducing the activity of acyl-Coenzyme A (CoA) and cholesterol acyltransferase in the liver of Zucker rats [118]. The study reported that novel cholesterol-lowering peptides derived from silver carp muscle demonstrated a dual cholesterol-lowering function, specifically by inhibiting the absorption of TC and promoting the uptake of peripheral LDL [93]. Goby undigested muscle proteins and thornback ray sarcoplasmic proteins had hypolipidemic activity [94,95]. In vivo studies on the anti-dyslipidemia peptide from Alaskan cod showed that the peptide could significantly regulate lipid metabolism and content in the liver and plasma, balance related liver gene expression, affect the liver fatty acid desaturase index, and promote fecal BA excretion and ileal BA transporter (IBAT) mRNA expression. In addition, Alaskan cod filet protein not only regulated the liver gene expression involved in lipid metabolism, but also regulated the expression of normal fat and high fat [96]. It has been reported that the presence of hydrophobic amino acids (Ile and Leu), acidic amino acids (Asp), and primary amino acids (His) in the peptide sequence are the reasons for the high antioxidant activity of fermented anchovies (Budu) [97]. When trypsin was used to enzymatically hydrolyze marine sea bass for 5 h, the degree of hydrolysis could reach 12.5% and the hypolipidemic active peptide was equivalent to that of the hypolipidemic drug cholestyramine [98]. Oyster peptide treatment inhibited PL activity in the intestines of high-fat diet (HFD)-fed mice, resulting in a reduction in their body weight, reduced visceral fat deposition, and improved dyslipidemia. Lipidomic analysis revealed that oyster peptide supplementation significantly decreased TG expression and increased glycerophospholipid expression [99]. The total lipid concentration in rats fed with scallop ovary peptide (6.1 mg/dL) was significantly lower than that of the control group (278.2 mg/dL) (*p* < 0.05), and the fecal excretion of BA was significantly increased [100].

### 4.2. Plant-Derived Anti-Hyperlipidemic Peptides

Soybean peptides (SPs), obtained through the enzymatic hydrolysis of soybean proteins, exhibit diverse biological activities, including lipid-lowering effects. SPs were able to significantly reduce the TC and TG levels in the blood and increase the levels of HDL-C. The potential mechanism may be related to inhibiting TC synthesis in the liver, promoting TC metabolism, and regulating the gene expression related to lipid metabolism. Specifically, the peptides IAVPGEVA, IAVPTGVA, and LPYP derived from soybean proteolysis were able to inhibit HMGCoAR activity and subsequently regulated TC metabolism in HepG2 cells. These peptides increased the ability of HepG2 cells to take up LDL through the activation of the LDL receptor/sterol regulatory element-binding protein 2 (LDLR-SREBP2) pathway. Moreover, these peptides were able to cross the intestinal barrier and be transported to the basolateral side, thereby exerting their biological activity. Further studies also revealed that the peptides FPFPRPPHQ, FMYL, MMLM, YSPHs, and SFFFPFELPRE derived from mature soybean proteolysates had potential cholesteryl esterase (CEase) inhibition effects [101].

LILPKHSDAD and LTFPGSAED are two specific lupin peptides that are efficiently taken up in the human colorectal adenocarcinoma cells Caco-2; computer simulation docking studies have revealed that these two peptides may have the potential to act as HMGCoAR inhibitors. Zanoni et al. confirmed the inhibitory effect of both peptides on HMGCoAR function through in vitro experiments and detailed their molecular mechanisms in regulating TC metabolism in HepG 2 cells. Specifically, both peptides were capable of using LDLR to significantly enhance the level of protein by inhibiting HMGCoAR activity. This increased level of LDLR allowed HepG 2 cells to absorb extracellular LDL molecules more efficiently, resulting in a decrease in intracellular TC levels. Furthermore, the intracellular processing of PCSK 9 was evaluated. The results showed that only the LILPKHSDAD peptide reduced the protein levels of PCSK 9 and hepatocyte nuclear factor 1 α (HNF 1-α), which subsequently led to a reduction in PCSK 9 secretion by HepG 2 cells. This finding further revealed the unique role of the LILPKHSDAD peptide in regulating TC metabolism and reducing TC levels [102].

Zanoni et al. employed high-performance liquid chromatography–electrospray ionization–tandem mass spectrometry (HPLC-ESI-MS/MS) to prepare hydrolyzed products of hemp seed protein, identifying 90 peptides. This hydrolysate exhibited a dose-dependent inhibition of the HMGCoAR catalytic activity in the concentration range of 0.1–1.0 mg/mL. Immunoblot analysis revealed that the HMGCoAR protein expression levels of SREBP2, LDLR, and pcsk9 were upregulated by the treatment of HepG 2 cells with 0.25, 0.5, and 1.0 mg/mL hydrolysates. However, the activation of the phosphorylated-5′-adenosine monophosphate-activated protein kinase (AMPK) signaling pathway was also observed, resulting in the inactivation of HMGCoAR via phosphorylation. Combined, the above data revealed the potential efficacy of hemp seed active peptide to reduce blood lipid levels, and its mechanism of action was similar to that of statins [119].

Nan et al. used ultrasound–microwave-assisted enzyme digestion technology to prepare hericium mushroom polypeptide (HEP). Three HEP components were successfully isolated with weights of 5 kDa and 10 kDa, named HEP-I (10.70% ± 1.21%), HEP-II (56.19% ± 1.06%), and HEP-III (33.11% ± 0.94%). The results showed that the HEP-III components showed significant antioxidant and hypolipidemic activity before and after gastrointestinal digestion, and the activity of the digested peptide components showed high gastrointestinal stability. In particular, the HEP-II components with molecular weights ranging from 5 to 10 kDa exhibited particularly prominent antioxidant and hypolipidemic capabilities. This may be attributed to the fact that the secondary structure of HEP-II is mainly composed of random coiling (18.36%) and α-helices (47.71%), which facilitate its antioxidant and hypolipidemic activities. Animal experiments further confirmed that HEP-II components had a significant lipid-lowering effect by improving blood lipid levels and enhancing liver antioxidant capacity. Combined with pathological observation and analysis, HEP-II components could improve liver fatty lesions and lipid metabolism disorders, reducing lipid accumulation in the liver so as to protect the liver and reduce the risk of fatty liver [105].

Chickpeas (CPes) belong to the legume family, and it was found that CPe-III peptide (RQSHFANAQP, 1155 Da) purified from the albumin hydrolysate of chickpea had an antioxidant activity, which was correlated with lipid metabolism and atherosclerosis. It was noted that the CPe-III peptide significantly decreased TC and TG levels in the serum and the accumulation of TC and TGs in the liver of HFD-fed mice, indicating that the CPe-III peptide was able to ameliorate hyperlipidemia. In addition, the CPe-III peptide was bound in the cavity of the TC transfer protein (CETP) to form four stable hydrogen bonds. Hydrophobic interactions were the main driving force in the binding process, and the binding of the CPe-III peptide to CETP suggested that CPE-III exerted a hypolipidemic role by blocking TC transport [106].

The peptide Glu-Phe-Leu-Glu-Leu-Leu (EFLELL) with significant activity was identified from rapeseed, and further experiments showed that rapeseed peptide treatment significantly reduced TC, TG, and LDL-C levels, while the changes in protein and gene expression levels of PCSK 9 and LDLR revealed the underlying mechanism. Molecular docking analysis showed that the intermolecular binding energies of the rapeseed peptide and LDLR-PCSK 9 were −6.3 kcal/mol and −8.1 kcal/mol, respectively. This study confirmed that the rapeseed peptide EFLELL exerted an active hypolipidemic effect by regulating the LDLR-PCSK 9 signaling pathway [107].

Ajayi et al. produced novel bioactive peptides with anti-hypercholesterolemic activity from quinoa protein hydrolysates (QPHs). It was found that only four bioactive peptides derived from QPHs were able to bind to the active site of CEase, while 12 peptides were bound to the active site of PL. The peptide sequences QHPHGLGALCAAPPST, HVQGHPALPGVPAHW, and ASNLDNPSPEGTVM were identified as potential CEase inhibitors. Meanwhile, based on the maximum number of reactive residues, FSAGGLP, QHPHGLGALCAAPPST, KIVLDSDDPLFGGF, MFVPVPH, and HVQGHPALPGVPAHW were identified as potential PL inhibitors [108]. The effect of walnut meal peptide (WMP) on the lipid metabolism of HFD-fed rats was investigated in a study by Yang et al. The experimental results showed that WMP intake effectively counteracted the HFD-induced increase in body weight and liver and epididymal fat weight, and decreased the serum levels of TC, TGs, and LDL-C, as well as liver TC and TG content. In addition, WMP led to an increase in HDL-C levels and a decrease in the atherosclerosis index (AI). Pathological section analysis revealed that WMP was able to reduce the hepatic steatosis and damage caused by the HFD. In addition, WMP supplementation normalized the HFD-induced increase in apoprotein (Apo)-B and the decrease in Apo-A1 levels, as well as causing favorable changes in the expression of lipid-metabolism-related genes (LCAT, CYP7A1, HMGR, and FAS) [109].

Amaranth, as a false grain, has a protein content of up to 13–19%. A series of bioactive peptides can be obtained through the hydrolysis of amaranth protein. These bioactive peptides play a key role in regulating lipid metabolism through their interactions with CEase and PL. Among the numerous identified bioactive peptides, the FPFVPAPT peptide was predicted to have potential CEase inhibitory activity, while peptides such as MPFLPR, FPFVGP, FPFPPTLGY, FGAPR, and FPFVPAPT were confirmed as PL inhibitors [103]. Among the cowpea active peptides, the GCTLN peptide was the only peptide that could significantly bind HMGCoAR, and the inhibitory rate was 47.8–57.1%. This inhibitory effect was achieved through the interaction of the GCTLN peptide with TC micelles containing phosphatidylcholine, which, in turn, reduced the solubility of the TC micelles [104].

### 4.3. Animal-Derived Anti-Hyperlipidemic Polypeptides

**Figure 4:**
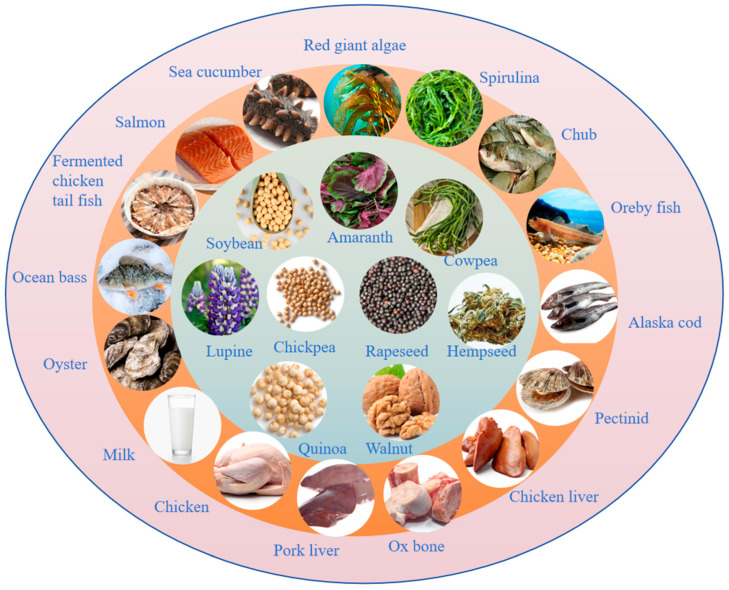
Sources of anti-hyperlipidemic peptides.

Animal-derived peptides are mainly extracted from animal tissues, blood, milk, and other sites (Figure 4). Protein-derived peptides isolated from bovine cheese, such as Leu-Gln-Pro-Glu (LQPE), Val-Leu-Pro-Val-Pro-Gln (VLPVPQ), and Val-Ala-Pro-Phe-Pro-Glu (VAPFPE), had anti-hyperlipidemic functions, all of which improved lipid metabolism disorders in hyperlipidemic mice, significantly reduced the levels of LDL-C and TGs in serum, and inhibited the development of hepatic lipid accumulation and the development of steatosis. VAPFPE reduced intestinal TC absorption and cholesterol ester formation by inhibiting the expression of hepatic cell nuclear factor 4 α (HNF 4 α), Nieman selected C1-like 1 (NPC1L1), and acetyl-CoA acetyltransferase 2 (ACAT 2), as well as increasing intestinal TC efflux by upregulating the ATP binding cassette transporter G8 (ABCG 8). VAPFPE maintained TC homeostasis by upregulating hepatic low-density lipoprotein receptor (LDL-R) expression [110]. Zhang et al. explored the effect of chicken collagen hydrolysate on atherosclerosis apolipoprotein E deficient C57BL/6. After 12 weeks of administration, the contents of TGs, TC, interleukin-6, soluble cell adhesion molecule-1, and tumor necrosis factor were significantly decreased in mice, indicating that chicken collagen hydrolysate could prevent and improve hyperlipidemia symptoms and inhibit the expression of inflammatory cytokines through its lipid-lowering effect [111]. Shimizu et al. studied the influence of pig liver protein hydrolysate on the lipid metabolism of genetically obese rats. After 14 weeks of continuous intake of pig liver protein hydrolysate, body weight and liver weight decreased significantly and the activities of glucose-6 phosphate dehydrogenase and fatty acid synthase also significantly reduced, indicating that pig liver protein hydrolysate regulated lipid metabolism in rats by inhibiting fat biosynthesis [112]. Fermented camel milk contains lactic acid bacteria, and the peptides produced by lactic acid bacteria had a TC-lowering effect because they can break down bile salts and prevent TC reabsorption in the gut. Experimental data showed that drinking camel milk for 45 days could significantly reduce TC, TGs, free fatty acids, and LDL in the plasma, liver, heart, and kidney [113]. Yang et al. demonstrated that chicken liver hydrolysates (CLHs) showed inhibitory lipase activity and BA-binding activity in vitro, and reduced serum/liver lipids and peroxidation, while improving serum TC levels and total antioxidant capacity in hamsters fed a HFD. In the expression of the enzyme genes involved in lipid homeostasis, CLH-treated hamsters exhibited downregulated adipogenesis, but upregulated energy expenditure and TC metabolism. CLHs showed similar relief effects on lipid metabolism and oxidative stress compared to the HFD-fed group supplemented with pure carnosine [114]. Cao et al. studied the effect and mechanism of bovine bone-gelatin-derived peptides (BGPs) on hypertension and hypertension-related complications in spontaneously hypertensive rats (SHRs). It was found that BGPs significantly reduced blood pressure, TGA levels, and the LDL-C/HDL-C ratio in SHRs by downregulating angiotensin-converting enzyme (ACE), angiotensin II (Ang II), and type Ang II 1 receptor (AT1R) levels, as well as upregulating Ang II 2 receptor (AT2R) levels. In brief, BGP could alleviate dyslipidemia in SHRs by inhibiting the ACE/Ang II/AT1R and activating the Ang II/AT2R signaling pathway [115]. The hypolipidemic effect of camel milk protein hydrolysate (CMPH) on streptozotocin-induced diabetic rats was demonstrated by the normalization of serum lipid levels, demonstrating the modulation of serum lipid levels after 8 weeks of CMPH administration [116].

## 5. Limitations of Peptides

The poor metabolic stability and low bioavailability of peptides greatly limit their development as clinically useful drugs. Firstly, poor metabolic stability means that peptides are easily decomposed and thus lose their pharmacological activity. This breakdown may occur in the digestive tract or in blood circulation, causing the peptide to fail before reaching the target tissue or organ. Secondly, low bioavailability means that even if the peptide can successfully enter the body, only a small part of it can be absorbed by the body and become effective. This may be due to factors such as the molecular structure, the charge nature of the peptide, or the interaction with other molecules. Moreover, the relatively short half-life of peptides in the body means that they are quickly metabolized soon after being absorbed by the body, limiting their sustained lipid-lowering effects. At the same time, different individuals also differ in their absorption of and response to peptides, which may lead to the inability to obtain the expected treatment effect after administration [120]. Therefore, despite the potential lipid-lowering effects, these limitations need to be overcome in practice to improve their efficacy and applicability.

## 6. Conclusions and Outlook

Hyperlipidemia is a chronic disease influenced by genetic factors, dietary habits, lifestyle choices, and other predisposing factors. Peptides play a role in alleviating hyperlipidemia by inhibiting cholesterol synthesis; promoting cholesterol excretion; regulating lipoprotein metabolism; and regulating TC, TG, BA, HDL, LDL, LDL, VL, IDL, and NF-κB levels through anti-inflammatory and antioxidant effects. These peptides are mainly derived from marine organisms, plants, and animals. Active anti-hyperlipidemia peptides could be used as potential candidates for hypolipidemic drugs. Despite their potential, the research and application of anti-hyperlipidemic peptides face challenges, including the complex and costly extraction and purification processes. Additionally, optimizing the bioavailability and stability of peptides remains a key challenge. Peptide metabolism in the human body is complex, and the efficacy and safety of peptides must be validated through large-scale clinical trials.

## Figures and Tables

**Table 1:** Source of peptides and mechanism of anti-hyperlipidemic action.

Polypeptide Source		Polypeptide Sequence	Anti-Hyperlipidemic Mechanism	Objects	Reference	Time
Ocean source	SCGH	Polypeptide mixtures	Eliminate BA	Rats	[88]	2022
	Spirulina	LDAVNR/MMLDF, C-phycocyanin	Prevent vascular disease	Cells	[89]	2013
	Chlorella	11-Peptides	Prevent chronic inflammation	Cells	[90]	2013
	Palmaria palmata	PAF-AH inhibitory peptide	Prevent atherosclerosis	Cells	[91]	2013
	Microalgae	Polypeptide mixtures	Reduce OX-LDL apoptosis	Rats	[92]	2022
	Chub	Polypeptide mixtures	Inhibit TC absorption Promote LDL uptake	Mice	[93]	2023
	Goby fish	Polypeptide mixtures	Reduce blood fat	Rats	[94]	2018
	Thornback	Polypeptide mixtures	Reduce blood fat	Rats	[95]	2018
	Alaska cod	Polypeptide mixtures	Promote fecal BA excretion and expression of IBAT mRNA levels	Pigs, Rats, Mice	[96]	2016
	Salmon	Polypeptide mixtures	Promote fecal BA excretion and expression of IBAT mRNA levels	Pigs, Rats, Mice	[96]	2016
	Fermented chicken tail fish	Polypeptide mixtures	Antioxidant		[97]	2018
	Ocean bass	Polypeptide mixtures	Reduce blood fat		[98]	2013
	Oyster	Polypeptide mixtures	Inhibit PL activity	Mice	[99]	2024
	Pectinid	Polypeptide mixtures	Promote fecal BA excretion	Rats	[100]	2014
Plant origin	Soybean	IAVPGEVA, IAVPTGVA, LPYP, FPFPRPPHQ, FMYL, MMLM, YSPHs, SFFFPFELPRE	Inhibit the activity of HMGCoAR, inhibit CEase	Cells	[101]	2015
	Lupine	LILPKHSDAD, LTFPGSAED	Inhibitor of the HMGCoAR, lower PCSK9	Cells	[102]	2017
	Amaranth	FPFVPAPT, MPFLPR, FPFVGP, FPFPPTLGY, FGAPR, FPFVPAPT	CEase inhibitor, PL inhibitor	Pigs	[103]	2021
	Cowpea	GCTLN	Inhibit the activity of HMGCoAR		[104]	2015
	Hempseed	Polypeptide mixtures	Inhibit the catalytic activity of HMGCoAR	Cells	[102]	2017
	Hericium erinaceus	Polypeptide mixtures	Improve the antioxidant capacity	Mice	[105]	2022
	Chickpea	RQSHFANAQP	Block TC transport	Mice	[106]	2018
	Rapeseed	EFLELL	Regulate the LDLR-PCSK9 signaling pathway	Cells	[107]	2023
	Quinoa	QHPHGLGALCAAPPST, HVQGHPALPGVPAHW, ASNLDNPSPEGTVM, FSAGGL, PQHPHGLGALCAAPPST, KIVLDSDDPLFGGF, MFVPVPH, HVQGHPALPGVPAHW	CEase inhibitor, PL inhibitor		[108]	2023
	Walnut	Polypeptide mixtures	Increase ApoB levels	Rats	[109]	2021
Animal origin	Milk	LQPE, VLPVPQ, VAPFPE	Inhibit HNF4α Increase intestinal TC efflux	Mice	[110]	2024
	Chicken	Polypeptide mixtures	Inhibit the expression of inflammatory cytokines	Mice	[111]	2010
	Pork liver	Polypeptide mixtures	Inhibit fat biosynthesis	Rats	[112]	2006
	Camel fermented milk	Polypeptide mixtures	Decompose bile salts		[113]	2024
	Chicken liver	Polypeptide mixtures	Inhibit LP activity and BA binding activity	Hamsters	[114]	2014
	Ox bone	Polypeptide mixtures	Inhibit ACE/AngII/AT1R Activate AngII/AT2R	Rats	[115]	2022
	Honey bee venom	Mellitin, apamin, secapin, tertiapin, ado lapin, MCD peptide	Increase insulin secretion in pancreatic β -cells		[116]	2018
